# Outcomes of Patients With Metastatic Differentiated Thyroid Cancer After Excellent Response to Treatment

**DOI:** 10.3389/fendo.2022.923182

**Published:** 2022-06-28

**Authors:** Chia-Jung Hsu, Kun-Yu Lai, Yu-Ling Lu, Ming-Hsien Wu, Feng-Hsuan Liu, Shu-Fu Lin

**Affiliations:** ^1^Division of Endocrinology and Metabolism, Department of Internal Medicine, Chang Gung Memorial Hospital, Taoyuan City, Taiwan; ^2^Division of Endocrinology and Metabolism, Department of Internal Medicine, New Taipei Municipal TuCheng Hospital (built and operated by Chang Gung Medical Foundation), New Taipei City, Taiwan; ^3^College of Medicine, Chang Gung University, Taoyuan City, Taiwan

**Keywords:** differentiated thyroid cancer (DTC), excellent response, distant metastasis (DM), risk factor, outcome

## Abstract

**Background:**

To evaluate the outcomes in differentiated thyroid cancer (DTC) patients who achieved excellent response to initial treatment and developed distant metastasis during follow-up.

**Methods:**

Thyroid cancer patients registered in Chang Gung Memorial Hospital thyroid cancer database between January 1979 and December 2019 were assessed.

**Results:**

Among 1053 DTC patients with excellent response to initial therapy, 14 (1.3%) patients developed metastatic disease during follow-up, including 6 males and 8 females with median age of 50.2 years [interquartile range (IQR), 39.9-53.7]. Nine (64.3%) patients had papillary cancer, four (28.6%) had follicular cancer, and one (7.1%) had Hürthle cell cancer. Most patients (92.9%) had stage I disease at diagnosis. The sites of metastasis were lung (71.4%), bone (7.1%), mediastinum (7.1%) and multiple sites (14.3%). With a median follow-up of 18.3 years (IQR, 14.8-23.8), 2 patients had disease-specific mortality. The 5- and 10-year disease-specific survival after the diagnosis of distant metastasis was 92% and 74%, respectively. Multiple sites of metastasis was associated with increased risk of mortality (P = 0.022).

**Conclusions:**

A small proportion of DTC patients with an excellence response to initial therapy developed distant metastasis during follow-up. Multiple organ distant metastases conferred a worse disease-specific survival.

## Introduction

Differentiated thyroid cancer (DTC), comprising of papillary (PTC), follicular (FTC) and Hürthle cell cancer (HCC) accounts for the majority (>90%) of all thyroid cancers (1). The incidence of DTC has increased in recent four decades worldwide (2, 3). Patients with DTC usually have a favorable prognosis with the 5-year survival rate > 97% according to the National Cancer Institute’s Surveillance, Epidemiology, and End Results Program Institute (4). However, recurrence of DTC is far more common than disease-related mortality with a 40-year recurrence rate up to 35% (5).

An initial risk stratification system is developed by the American Thyroid Association (ATA) to estimate the risk of recurrence based on pathological characteristics, lymph node involvement, and the presence of genetic mutations after primary surgery (6). In addition, a dynamic risk stratification system is applied to estimate the ongoing risk of recurrent disease during follow-up (6–8). The response to therapy is divided into four categories: excellent response, biochemical incomplete response, structural incomplete response and indeterminate response. Patients with excellent response have a low recurrence rate (1-4%) and low mortality rate (<1%) (8). Therefore, a decrease in the intensity and frequency of follow-up is recommended for patients who have excellent response to initial therapy (9).

Long-term follow-up studies reveal that the recurrence rate was between 1.0% and 2.8% in DTC patients who had excellence response to initial treatment during a median follow-up of 4-10.4 years (9–13). In these studies, most patients (88.7%) with recurrence were biochemical disease or local recurrence. However, distant metastasis occurred in the remaining patients (11.3%). Of note, the mortality rate was up to 37.5% in patients who developed distant metastasis (13). These results demonstrated that a small proportion of DTC patients with excellent response to initial treatment would develop fatal metastatic disease during follow-up. The risk factors associated with mortality in these patients with distant metastasis were not addressed in that study and needs to be clarified.

In this study, we evaluated the clinical course and risk factors associated with mortality in DTC patients who had excellent response to initial treatment and developed distant metastasis during follow-up.

## Materials and Methods

### Data Source

Data in a thyroid cancer database at Chang Gung Memorial Hospital in Linkou, Taiwan were analyzed. This database was established with the intent to enhance research on thyroid cancer (14, 15). Information of thyroid cancer patients managed at this tertiary care hospital was prospectively gathered. Clinical characteristics, imaging data, laboratory findings, pathological results, treatment, and clinical course were rigorously recorded.

### Study Cohort

A total of 5023 patients were identified in this database between January 1979 and December 2019. Patients were excluded if they met the following exclusion criteria: did not receive total thyroidectomy, histologic types were not DTC, had distant metastasis at diagnosis, did not reach undetectable thyroglobulin (Tg) during the first 2 years after initial treatment, did not have 3 consecutive results of undetectable Tg, the presence of thyroglobulin antibody (TgAb), lack of TgAb data throughout the clinical course. Tg was measured under TSH suppression in this study.

### Treatment Protocol and Follow-Up

Most DTC patients received total thyroidectomy when tumor size was > 1 cm (before 2016) or > 2 cm (since 2016). Neck lymph node dissection was performed for apparent nodal metastasis. Radioactive iodine (RAI) was administered for patients with intermediate and high risk for recurrence (6, 16, 17). After initial treatment, levothyroxine was administered for thyroid hormone replacement or thyrotropin suppression. Measurements of Tg and TgAb levels and neck ultrasonography (US) were generally performed every 3-12 month following initial therapy (6). Serum Tg measurements were conducted using immunoradiometric assay kit (CIS Bio International, Paris, France) before 2014 and the indicated functional sensitivity was at least 1 ng/mL. From August 2014, the measurement switched to sandwich enzyme-linked immunosorbent assay (Beckman Coulter, Inc., Minnesota, U.S.A.) with an indicated functional sensitivity at least 0.1 ng/mL. TgAb was measured using radioimmunoassay (Perkin Elmer, Massachusetts, U.S.A.) between 1979 and 2010 and changed to electrochemiluminescence immunoassay (Roche Diagnostics GmbH - Mannheim, Germany) from May 2011 with a threshold of 115 IU/mL. These tests were conducted in a College of American Pathologists accredited laboratory at Chang Gung Memorial Hospital, Linkou, Taiwan.

Imaging studies, including RAI scan, computerized tomography (CT), 18F-fluorodeoxyglucose positron emission tomography/computed tomography (18F-FDG PET/CT), and Tc-99m methylene diphosphonate whole body bone scan were performed with a suspicion of recurrence. Histological diagnosis of distant metastasis was made when indicated.

### Definitions

An excellent response was defined as undetectable Tg for at least three consecutive tests, without the presence of TgAb, and without suspicious lesions on physical examination, neck US or other imaging studies when indicated. Patients were categorized according to the 8^th^ edition American Joint Committee on Cancer (AJCC) staging system and the ATA risk stratification system (low, intermediate, and high risk of recurrence) (6, 18).

### Ethics Statement

The Ethics Committee of the Institutional Review Board at Chang Gung Memorial Hospital approved this study (No. 202101951B0), and the requirement to obtain informed consent was waived. This study was conducted in accordance with the Declaration of Helsinki.

### Statistical Analysis

Continuous variables without normal distribution were expressed as median and interquartile range (IQR). Categorical variables were presented as number and percentage. The comparisons of the characteristics were calculated using Fisher’s exact test and Mann-Whitney U test. Kaplan-Meier method was used to estimate disease-specific survival (DSS) and cumulated incidence of distant metastasis. Log-rank test was used to determine the 5-, 10- and 20-year DSS rates. Statistical analyses were performed using SPSS statistical software (version 24.0, SPSS Inc.). P < 0.05 was considered statistically significant.

## Results

### Study Cohort

**Figure 1:**
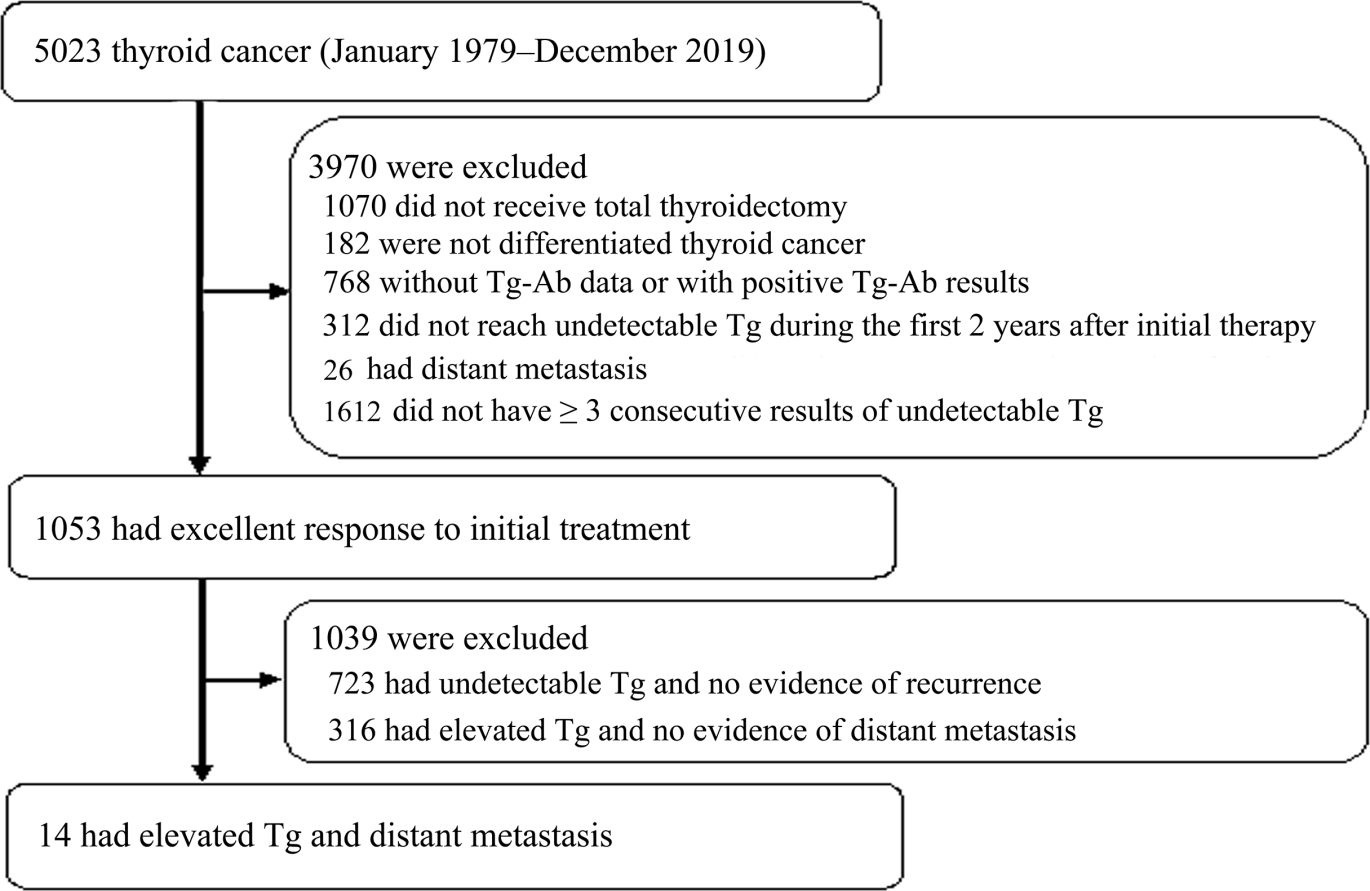
Flowchart of cohort establishment.

A total of 5023 thyroid cancer patients were identified in the dataset between January 1979 and December 2019 (**Figure 1**). Patients were excluded when they met the exclusion criteria: 1070 did not receive total thyroidectomy, 182 were not DTC, 768 did not have TgAb data or had positive results of TgAb, 312 did not reach undetectable Tg during the first 2 years to initial therapy, 26 had distant metastasis upon the diagnosis of thyroid cancer and 1612 did not have ≥ 3 undetectable Tg levels after initial treatment. There were 1053 patients achieved excellent response to initial treatment. During follow-up, 723 had undetectable Tg and no evidence of recurrence, 316 had elevated Tg and no evidence of distant metastasis, and 14 patients had detectable Tg and distant metastasis. These 14 patients with distant metastasis were included for further analysis.

### Baseline Characteristics of Patients With Distant Metastasis

The baseline characteristics of 14 patients with distant metastasis are shown in **Table 1**. The median age of these patients was 50.2 years (IQR, 39.9–53.7 years), and 8 (57.1%) patients were female. The majority (64.3%) of patients had PTC (6 with classical PTC and 3 with follicular variant of PTC), followed by FTC (28.6%) and HTC (7.1%). The median tumor size was 3.8 cm (IQR, 2.5–4.4 cm). Most patients had tumors larger than 4 cm (42.9%), followed by 2–4 cm (35.7%) and ≤ 2 cm (21.4%). Most patients had T4 stage (42.8%), followed by T3 stage (28.6%), T2 stage (14.3%), and T1 stage (14.3%). 3 DTC tumors (21.4%) had capsular invasion and 3 (21.4%) had vascular invasion. There was no evidence of cervical lymph node metastasis or distant metastasis in all of these patients after initial treatment. According to the 8^th^ TNM staging system, most (92.9%) patients had stage I disease and the remaining patient (7.1%) had stage II disease. Based on the ATA initial risk stratification, 50.0%, 7.1%, and 42.9% patients were classified as low risk, intermediate risk, and high risk, respectively. All patients underwent total thyroidectomy. Twelve patients (85.7%) received RAI for thyroid remnant ablation. RAI remnant uptake was measured in 10 patients and the median remnant uptake was 0.63% (IQR, 0.40–1.11). The median cumulative dose of RAI prior to metastasis was 160.0 mCi (IQR, 90.0–260.0 mCi). All patients had detectable Tg prior to the diagnosis of metastasis, with the median level of first detectable Tg of 1.56 ng/mL (IQR, 0.78–2.66 ng/mL). The median duration between undetectable and detectable Tg was 10.2 years (IQR, 6.4–15.1 years), and the median Tg level before the diagnosis of metastasis was 4.41 ng/mL (IQR, 1.5–10.5 ng/mL). It took a median time of 1.8 year (IQR, 1.3–2.4 years) between the detection of elevated Tg level and the diagnosis of distant metastasis. During a median follow-up of 12.0 years (IQR, 8.2–16.8 years), these patients were proven to have metastatic disease at the median age of 60.4 years (IQR, 53.5–73.1 years). The most common metastatic site was lung (71.4%), followed by multiple sites (14.3%), bone (7.1%), and mediastinum (7.1%). The median follow-up period was 18.3 year (IQR, 14.8–23.8 years) in this study.

**Table 1:** Clinical features of 14 patients with excellent response after initial therapy and developed metastatic disease during follow-up.

Characteristic	Patients (n=14)
Age at diagnosis, years (IQR)	50.2 (39.9, 53.7)
Gender, n (%)	
Male	6 (42.9%)
Female	8 (57.1%)
Histological type, n (%)	
Papillary*	9 (64.3%)
Follicular	4 (28.6%)
Hürthle cell	1 (7.1%)
Tumor size, cm (IQR)	3.8 (2.5, 4.4)
Tumor size, n (%)	
≤2 cm	3 (21.4%)
>2-4 cm	5 (35.7%)
>4 cm	6 (42.9%)
T stage, n (%)	
T1	2 (14.3%)
T2	2 (14.3%)
T3	4 (28.6%)
T4	6 (42.8%)
Lymph node metastases, n (%)	
N0	14 (100%)
N1	0
Distant metastases, n (%)	
M0	14 (100%)
M1	0
TNM stage at diagnosis, n (%)	
I	13 (92.9%)
II	1 (7.1%)
III	0
IV	0
ATA risk level, n (%)	
Low	7 (50.0%)
Intermediate	1 (7.1%)
High	6 (42.9%)
Operation method, n (%)	
Total thyroidectomy	14 (100%)
RAI remnant ablation, n (%)	
Yes	12 (85.7%)
No	2 (14.3%)
Cumulative dose of RAI therapy before metastasis, mCi (IQR)	160.0 (90.0, 260.0)
Time from DTC diagnosis to metastasis, years (IQR)	12.1 (8.2, 16.8)
Age at metastasis, years (IQR)	60.4 (53.5, 73.1)
Sites of metastases, n (%)	
Lung	10 (71.4%)
Bone	1 (7.1%)
Mediastinum	1 (7.1%)
Multiple (lung and bone)	2 (14.3%)
First detectable Tg level, ng/mL (IQR)	1.56 (0.78, 2.66)
Time between undetectable and detectable Tg, years (IQR)	10.2 (6.4, 15.1)
Latest detected Tg level before metastasis confirmed, ng/mL (IQR)	4.41 (1.53, 10.50)
Time between detectable Tg and metastasis confirmed, years (IQR)	1.8 (1.3, 2.4)
Follow-up, years (IQR)	18.3 (14.8, 23.8)

Tg, thyroglobulin; RAI, radioactive iodine; IQR, interquartile range.

*Including 6 classical subtype and 3 follicular variant of papillary thyroid cancer.

The characteristics and clinical course of these patients are summarized in **Table 2**. Patients 1 and 11 achieved no evidence of disease (undetectable Tg and negative findings on diagnostic RAI scan) after total thyroidectomy. RAI treatment was not performed in these 2 patients following primary surgery. Distant metastasis was identified using RAI scan (14.3%), CT (28.6%), 18F-FDG PET/CT (50.0%), Tc-99m methylene diphosphonate bone scan (7.1%), and histologic examination (57.1%). Additional treatments for these patients included external beam radiation therapy (n=3), lenvatinib (n=2) and sorafenib (n=2). Among these 14 patients, 2 (14.3%) died due to DTC and the remaining 12 (85.7%) patients were alive when this study was closed.

**Table 2:** Details of 14 patients with distant metastasis.

No	Gender/Age^*^	Histology	Initial TNM	Initial stage	ATA risk	Cumulative dose of RAI before metastasis (mCi)	Time to metastasis (year)	Positive findings at detection	Sites of metastasis	Cumulative dose of RAI (mCi)	First detectableTg (ng/mL)	Latest detected Tg level before metastasis confirmed (ng/mL)	Follow up (year)	Additional therapy	Survival
1	F/37	Papillary	T4aN0M0	I	High	6	16.8	PET/CT, pathology	Lung	356	0.17	0.19	19.8		Y
2	F/38	Papillary	T1bN0M0	I	Low	130	7.0	Radioiodine scan	Lung	260	3.04	4.14	16.9		Y
3	M/40	Hürthle	T3aN0M0	I	Intermediate	190	8.2	PET/CT, pathology	Bone	640	0.78	6.27	12.9	Radiation therapy	Y
4	M/44	Papillary	T4aN0M0	I	High	260	9.8	PET/CT	Lung	530	1.42	4.30	12.9	Lenvatinib	Y
5	M/48	Papillary	T4aN0M0	I	High	120	7.1	Radioiodine scan	Mediastinum	220	0.11	0.32	7.5		Y
6	F/49	Follicular	T4aN0M0	I	High	100	15.5	CT, pathology	Lung	330	19.70	49.40	31.3		Y
7	F/50	Papillary	T2N0M0	I	Low	30	7.5	CT, pathology	Lung	590	2.14	10.50	20.3		Y
8	F/51	Papillary	T4aN0M0	I	High	400	23.8	PET/CT	Lung	400	3.75	4.52	28.8	Sorafenib	Y
9	F/54	Papillary	T4aN0M0	I	High	510	23.2	PET/CT	Lung	510	2.05	20.76	28.3	Lenvatinib, sorafenib	Y
10	M/54	Follicular	T3aN0M0	I	Low	235	19.5	CT, pathology	Lung	535	0.93	1.53	23.8		Y
11	M/58	Follicular	T3aN0M0	II	Low	6	13.0	CT, pathology	Lung	806	2.66	3.80	20.8		Y
12	M/66	Papillary	T2N0M0	I	Low	520	11.3	PET/CT, pathology	Lung	880	0.15	1.19	15.1		Y
13	F/35	Follicular	T3aN0M0	I	Low	90	10.2	PET/CT	Lung, bone	970	1.43	37.60	15.5	Radiation therapy	N
14	F/50	Papillary	T1bN0M0	I	Low	200	12.8	Bone scan, pathology	Lung, bone	410	1.69	7.66	14.8	Radiation therapy	N

*Age at diagnosis.

RAI, radioactive iodine; Tg, thyroglobulin.

### Risk Factors Associated With Disease-Specific Mortality in Patients With Metastasis

We sought to identify the risk factors correlated with disease-specific mortality in 14 patients who developed distant metastasis (**Table 3**). Due to small sample size (n=14), Fisher’s exact test and Mann-Whitney U test were used for the analysis of categorical data and continuous data, respectively. Fisher’s exact test and Mann-Whitney U test are considered appropriate in the examination of data with small sample size (19). Statistical analyses reveal that multiple sites (lung and bone) metastasis was significantly associated with disease-specific mortality (P = 0.022), while age, gender, histological type, tumor size, T stage, TNM stage, ATA risk level, thyroid remnant ablation, RAI cumulative dose, time from DTC diagnosis to metastasis, age at metastasis, first detectable Tg level, time between undetectable and detectable Tg, the latest detectable Tg level before metastasis confirmed, time between detectable Tg and metastasis confirmed, and cumulative RAI dose throughout the clinical course did not reveal any significant association with mortality. The number of patients was limited that precluded multivariate analysis.

**Table 3:** Risk factors associated with disease-specific mortality in 14 patients with distant metastasis.

Variable	Mortality (n=2)	Survival (n=12)	*P* value
Age at diagnosis, years (IQR)	42.5 (34.6, 50.3)	50.8 (41.8, 53.9)	0.273
Gender, n (%)			
Male	0	6 (50.0%)	0.473
Female	2 (100%)	6 (50.0%)	
Histological type, n (%)			1.000
Papillary	1 (50.0%)	8 (66.7%)	
Follicular	1 (50.0%)	3 (25.0%)	
Hürthle cell	0	1 (8.3%)	
Tumor size, cm (IQR)	3.3 (1.9, 4.7)	3.8 (2.5, 4.4)	1.000
Tumor size, n (%)			0.769
≤2 cm	1 (50%)	2 (16.6%)	
>2-4 cm	0	5 (41.7%)	
>4 cm	1 (50%)	5 (41.7%)	
T stage, n (%)			0.308
T1	1 (50%)	1 (8.3%)	
T2	0	2 (16.7%)	
T3	1 (50%)	3 (25.0%)	
T4	0	6 (50.0%)	
Lymph node metastases, n (%)			–
N0	2 (100%)	12 (100%)	
N1	0	0	
Distant metastases, n (%)			–
M0	2 (100%)	12 (100%)	
M1	0	0	
TNM stage at diagnosis, n (%)			
I	2 (100%)	11 (91.7%)	1.000
II	0	1 (8.3%)	
III	0	0	
IV	0	0	
ATA risk level, n (%)			0.538
Low	2 (100%)	5 (41.7%)	
Intermediate	0	1 (8.3%)	
High	0	6 (50.0%)	
Operation method, n (%)			–
Total thyroidectomy	2 (100%)	12 (100%)	
RAI remnant ablation, n (%)			1.000
Yes	2 (100%)	10 (83.3%)	
No	0	2 (16.7%)	
Cumulative dose of RAI therapy before metastasis, mCi (IQR)	145.0 (90.0, 200.0)	160.0 (65.0, 330.0)	0.715
Time from DTC diagnosis to metastasis, years (IQR)	11.5 (10.2, 12.8)	12.1 (7.8, 18.1)	0.855
Age at metastasis, years (IQR)	54.0 (44.8, 63.1)	61.3 (53.8, 74.2)	0.361
Sites of metastases, n (%)			0.022
Lung	0	10 (83.3%)	
Bone	0	1 (8.3%)	
Mediastinum	0	1 (8.3%)	
Multiple (lung and bone)	2 (100%)	0	
First detectable Tg level, ng/mL (IQR)	1.56 (1.43, 1.69)	1.74 (0.48, 2.85)	1.000
Time between undetectable and detectable Tg, years (IQR)	9.8 (8.1, 11.5)	10.8 (5.7, 16.4)	0.855
Latest detected Tg level before metastasis confirmed, ng/mL (IQR)	22.63 (7.66, 37.60)	4.22 (1.36, 8.39)	0.144
Time between detectable Tg and metastasis confirmed, years (IQR)	1.6 (1.3, 2.0)	1.6 (0.9, 2.8)	0.714
Cumulative dose of RAI therapy, mCi (IQR)	690.0 (410.0, 970.0)	520.0 (343.0, 615.0)	0.361
Follow-up period, years (IQR)	15.2 (14.8, 15.5)	20.0 (14.0,26.0)	0.361

Tg, thyroglobulin; RAI, radioactive iodine; IQR, interquartile range.

### Disease-Specific Survival

**Figure 2:**
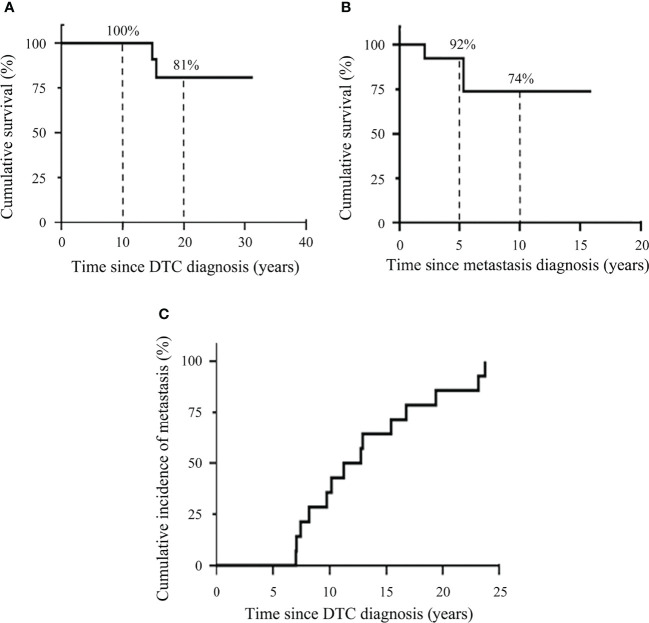
Kaplan–Meier plots demonstrating disease-specific survival of DTC patients and cumulative incidence of distant metastasis. **(A)** Kaplan–Meier plots for survival after the diagnosis of DTC. **(B)** Kaplan–Meier plots for survival after the diagnosis of distant metastasis. **(C)** Cumulative incidence of distant metastasis.

DSS after the diagnosis of DTC and DSS after the diagnosis of distant metastasis were analyzed using the Kaplan–Meier method with the log rank test. The 10-year and 20-year DSS after the diagnosis of DTC were 100% and 81%, respectively (**Figure 2A**). The 5-year and 10-year DSS after the detection of metastasis were lower with 92% and 74%, respectively (**Figure 2B**).

### Cumulative Incidence of Distant Metastasis

Cumulative incidence of distant metastasis was assessed using the Kaplan-Meier method (**Figure 2C**). The cumulative incidence of distant metastasis was at a median time of 12.1 years (range, 7.0-23.8 years).

### Risk Factors Associated With Distant Metastasis

We sought to identify the risk factors associated with distant metastasis between 723 patients who remained with excellent response during follow-up and 14 patients who had distant metastasis (**Table S1**). Univariate Cox regression analysis revealed that the risk of distant metastasis was significantly associated with male gender [hazard ratio (HR), 4.15; 95% confidence interval (CI), 1.44-11.97; P = 0.009] and larger tumor size (> 4 cm, HR, 7.47; 95% CI, 1.86-29.96; P = 0.005). In a multivariate Cox regression analysis adjusted for age, gender, histological type, tumor size and TNM stage at diagnosis, male gender (adjusted HR, 3.32; 95% CI, 1.13-9.80; P = 0.030) and larger tumor size (> 4 cm, adjusted HR, 4.71; 95% CI, 1.07-20.80; P = 0.041) were still significantly associated with higher risk of distant metastasis.

## Discussion

This study demonstrates that a small proportion (1.3%) of DTC patients who were found to have an excellent response to initial treatment would develop distant metastasis during a median follow-up of 18.3 years. All of these metastases were identified following the detection of rising levels of Tg. The majority (64.3%) of metastasis was detected more than 10 years after initial therapy. These findings suggest that long-term follow-up of Tg in patients with excellent response is needed. A similar study reveals 1.1% of DTC patients with excellent response to initial therapy developed metastatic disease during a median follow-up of 10 years (9). The most common metastatic site was lung in our (71.4%) and that (75.0%) studies.

In this study, all metastatic diseases were implicated by increasing levels of Tg. Further imaging evaluations, including neck US, RAI scan, CT, 18F-FDG PET/CT, and bone scan were performed for the detection of recurrent disease. Only one patient had local recurrence prior to the occurrence of metastatic disease. These findings highlight the importance of additional imaging studies for patients who have detectable Tg levels and negative neck US findings during follow-up. The ATA guidelines suggest neck and upper chest CT or MRI is needed for DTC patients with high Tg and negative neck US, and 18F-FDG PET/CT scanning is required for patients with elevated serum Tg and negative RAI imaging (6).

A prior report shows the recurrence after 5-year of follow-up occurs only in a small proportion (0.26%) of DTC patients with an excellent response to initial treatment (13). Therefore, a looser follow-up strategy for low-risk patients is suggested: annually for 5 years, then every 18-24 month. Our data reveal low-risk patients developed distant metastasis spanning from 7.0 to 23.8 years during the follow-up of DTC. These data indicate cautious and long-term follow-up for these patients is mandatory.

A recent study reveals that the mortality rate was 37.5% in DTC patients with excellent response to initial treatment but developed metastasis during follow-up (9). The mortality rate was lower (14.3%) in our study. Multi-organ distant metastasis was a significant risk factor for DSS. Our data indicate multi-organ metastasis is the most important prognostic factor for mortality in patients with metastatic DTC. This finding is consistent with a prior report showing multi-organ metastases confer poor DSS (20).

In this study, we found the median time from elevated Tg to the diagnosis of distant metastasis was 1.8 years. This data is similar with a study showing the median time of 1.5 year between elevated Tg and metastasis detection (9). Two of our 14 patients had low initial detectable Tg levels with a slowly increase before the diagnosis of distant metastasis (0.17 to 0.19 ng/mL and 0.11 to 0.32 ng/mL, respectively). The slightly raised Tg levels led to the diagnosis of distant metastasis in these patients.

The 2015 ATA guidelines suggest TSH level should be maintained at the low reference range (0.5–2 mU/L) in patients with excellent response to therapy (6). In our study, the median TSH level was 0.17 µIU/mL (IQR, 0.03–0.91) during the period of excellent response in these patients. Most of our patients (92.3%) had TSH levels within this recommendation range. Some prior reports failed to demonstrate the association between the degree of TSH suppression and DTC recurrence, indicating the ATA recommendation to maintain TSH to reduce the recurrence rate after initial treatment remains uncertain (21, 22).

A prior report demonstrates DTC patients with distant metastasis diagnosed during pre-surgery work-up had shorter survival than those with metastases detected by RAI scan, implicating the importance of detection timing on survival (23). In addition, a retrospective study analyzed 140 DTC patients with distant metastasis, the authors found most patients (65.8%) were categorized into low or intermediate risk of recurrence by using 2015 ATA guidelines. This study suggests the risk classification of 2015 ATA guidelines was inappropriate in these patients (24).

This study has strengths. This dataset contains a large cohort of thyroid cancer patients with a long-term follow-up that allows us to evaluate the long-term clinical outcomes of DTC patients who had excellent response to initial treatment.

This study exhibits some limitations. First, ATA’s guidelines recommend each Tg test have TgAb measured simultaneously. In this study, not all Tg tests had TgAb measured concurrently. A recent report indicates that 0.7% of patients with low-risk DTC had TgAb (25). A study indicates 11.7% of *de novo* TgAb appeared during the follow-up of DTC patients who were TgAb-negative at baseline. Comparable disease-free survival was observed in patients with *de novo* TgAb compared with controls (26). Second, we did not analyze the genetic alterations in this study. *CDKN2A* loss was associated with poor disease-specific survival in patients with advanced DTC (27). Third, due to the nature of its retrospective design, no consistent treatment and follow-up protocols existed in these DTC patients. All patients included in this study were followed at a single institute that might ensure a similar therapeutic approach and follow-up strategy.

## Conclusion

DTC patients with an excellent response to initial therapy have a low risk of distant metastasis during follow-up. Distant metastasis can occur more than 20 years after the diagnosis of DTC. A life-long follow-up with measurement of Tg to low-risk DTC patients is recommended. Multi-organ metastasis confers increased risk of mortality in these patients.

## Data Availability Statement

The original contributions presented in the study are included in the article/**Supplementary Material**. Further inquiries can be directed to the corresponding authors.

## Ethics Statement

The studies involving human participants were reviewed and approved by Institutional Review Board of Chang Gung Medical Foundation (No. 202101951B0). Written informed consent for participation was not required for this study in accordance with the national legislation and the institutional requirements.

## Author Contributions

C-JH and S‐FL contributed to the conception and design of the study. C-JH, K-YL, Y-LL, and M-HW acquired and analyzed the data. C-JH, K-YL, Y-LL, M-HW, F-HL, and S‐FL interpreted the data. C-JH wrote the manuscript. All authors reviewed and revised the manuscript.

## Funding

This study was supported by the New Taipei Municipal TuCheng Hospital (built and operated by Chang Gung Medical Foundation), New Taipei City, Taiwan (CORPVVL0041).

## Conflict of Interest

The authors declare that the research was conducted in the absence of any commercial or financial relationships that could be construed as a potential conflict of interest.

## Publisher’s Note

All claims expressed in this article are solely those of the authors and do not necessarily represent those of their affiliated organizations, or those of the publisher, the editors and the reviewers. Any product that may be evaluated in this article, or claim that may be made by its manufacturer, is not guaranteed or endorsed by the publisher.
