# Supplementary material for: Outcomes of Patients With Metastatic Differentiated Thyroid Cancer After Excellent Response to Treatment
Source: Front Endocrinol (Lausanne). 2022 Jun 28;13:923182. doi: 10.3389/fendo.2022.923182 (PMC9273717; doi:10.3389/fendo.2022.923182)
Supplement: Supplementary file 1 [file Table_1.docx]

Table S1. Risk factors associated with distant metastasis for patients with an excellent response to initial therapy.

|  | Univariate analysis | | |  | Multivariate | | |
| --- | --- | --- | --- | --- | --- | --- | --- |
|  | HR | 95% CI | *p* value |  | Adjusted HR^a^ | 95% CI | *p* value |
| Age at diagnosis (years) |  |  |  |  |  |  |  |
| < 55 | Reference | | |  | Reference | | |
| ≥ 55 | 1.08 | 0.24-4.82 | 0.923 |  | 0.95 | 0.12-7.91 | 0.965 |
| Gender |  |  |  |  |  |  |  |
| Female | Reference | | |  | Reference | | |
| Male | 4.15 | 1.44-11.97 | 0.009 |  | 3.32 | 1.13-9.80 | 0.030 |
| Histological type |  |  |  |  |  |  |  |
| Papillary | Reference | | |  | Reference | | |
| Follicular | 3.03 | 0.93-9.91 | 0.066 |  |  |  |  |
| Hürthle cell | 5.46 | 0.69-43.42 | 0.108 |  | 1.78 | 0.52-6.16 | 0.361 |
| Tumor size (cm) | 1.48 | 1.08-2.01 | 0.013 |  | 3.36 | 0.38-29.50 | 0.275 |
| Tumor size |  |  |  |  |  |  |  |
| ≤ 2 cm | Reference | | |  | Reference | | |
| > 2 to 4 cm | 2.11 | 0.50-8.84 | 0.307 |  | 1.84 | 0.43-7.99 | 0.414 |
| > 4 cm | 7.47 | 1.86-29.96 | 0.005 |  | 4.71 | 1.07-20.80 | 0.041 |
| TNM stage at diagnosis |  |  |  |  |  |  |  |
| I | Reference | | |  | Reference | | |
| II, III | 1.48 | 0.19-11.35 | 0.705 |  | 1.34 | 0.08-23.15 | 0.840 |

HR, hazard ratio; CI, confidence interval

^a^ Adjusted for age, gender, histological type, tumor size and TNM stage at diagnosis
